# Epidemiology of Accidents in Tile Factories of Mangalore City in Karnataka

**DOI:** 10.4103/0970-0218.62567

**Published:** 2010-01

**Authors:** S Ganesh Kumar, UP Rathnakar, HN Harsha Kumar

**Affiliations:** Department of Preventive and Social Medicine, JIPMER, Puducherry, India; 1 Department of Pharmacology, KMC Mangalore, Manipal University, India; 2 Department of Community Medicine, KMC Mangalore, Manipal University, India

**Keywords:** Epidemiology, accidents, tile factories, occupational safety

## Abstract

**Background:**

Occupational accidents are a major point of concern in industries. The academic community should take the first step to address the long-neglected concerns of occupational safety.

**Objective:**

To assess the prevalence and pattern of occupational accidents.

**Materials and Methods:**

A record-based, cross-sectional study was done in three tile factories of Mangalore city, in Karnataka. A total of 416 workers were analyzed for the year 2004, and data regarding age, sex, job duration, type and nature of injury, body parts involved, and time of injury were collected in a prestructured proforma.

**Statistical Analysis:**

Proportions, Chi-square test, Univariate and Multivariate analysis.

**Results:**

The overall prevalence rate of accidents was found to be 18.5%. It was found that almost around 86% of the accidents had affected the limbs (upper limb 24.7%, lower limb 61%), around half (52%) of the injuries were contributed by superficial injuries, 40% of accidents were due to stepping/striking against objects and while handling. Hand tools and machinery in motion contributed to around 20% of the accidents. Accidents were more common among the younger age group and less-experienced workers. Multiple logistic regression analyses revealed that the age group of 30-39 years had an independent significant association with accidents (OR = 0.21, *P* = 0.04).

**Conclusion:**

Accidents in tile industries are an important occupational health problem in this area of the country. There is a need for proper safety training of the workers.

## Introduction

Traditionally labor-oriented markets are making a change toward more automation and mechanization, although a general awareness about occupational safety and occupational and environmental hazards were not spread in the society simultaneously.(1) Occupational accidents, although definitions vary widely, are those occurring at the place of work. In the agricultural sector, where more than half of the total workforce is employed, the prevalence rate of accidents ranges from 7.8 % to 27.7%, depending upon the type of work performed.(2) Prevalence of work-related injuries is 35% among all injuries reported in a public sector industry.(3)

Data on occupational accidents are not available from all countries in the world. The rates are different for individual countries and regions and for separate branches of economic activity. In 1994, at the global level an average estimated fatal occupational accident rate was found to be fourteen out of one lakh workers.(4) Although the profile of the at-risk worker population has changed greatly over the past decade, both quantitatively and qualitatively, the risk of occupational injury still centers on workers of various industries.(5)

The academic community should take the first step, in demonstrating its leadership, by addressing the long-neglected concerns of occupational safety. It is their primary concern to look after health promotion, specific protection, and safety measures of the workers. The medical fraternity has systematically ignored the importance of occupational health and safety in teaching, training, and epidemiological research. The government has not been playing a proactive role. Also, unemployment and poor hygienic conditions prevalent in the society, along with illiteracy and ignorance has further compounded the problem.(6) Employment injury statistics provide the essential information for Government Labor Inspectorates, the ESI scheme, and other agencies dealing with occupational safety. However, there are no published reports on the aspects of injury occurrence in tile industries in India. Studies of such a nature will be useful tools in developing appropriate prevention methods. With this background, the present study has been undertaken, to assess the prevalence and pattern of accidents in the tile factories of Mangalore city, Karnataka.

## Materials and Methods

This study was undertaken in three tile factories in Mangalore city, in the Dakshina Kannada district of Karnataka. The Dakshina Kannada district was known as a hub of industries in the state. The district is known for its tile industry, cashew factory, and beedi factory. However, the tile industry in Mangalore was once a major contributor to the economy of this region. From 36 factories in 1969, today there are only six tile factories. Out of these, three tile factories were selected by a simple random technique. A complete analysis of occupational injury records for the year 2004 was carried out. A total of 416 workers were analyzed.

Accident registers were examined for the study period, first to collect baseline data that included age, sex, and year of experience. To understand the nature of injury and the consequences of it, and also to facilitate the understanding of the cascade of events leading ultimately to injury, every single accident was analyzed according to the type of injury and the incident that led to the accident, as also the body parts involved. The data were collected as per the guidelines of International Labor Organization (ILO).(7) Whenever there was a doubt, the employer was contacted for clarification.

Univariate and Multivariate analyses were carried out for factors that included age, sex, and duration of experience. The findings were described in terms of proportions. Chi-square test was carried out to test the differences between proportions. Probability level of less than 0.05 was considered significant.

## Results

Age-wise distribution of workers showed that around 75% of the workers (310) were in the 30-49 years age range. About 18% of the subjects (75) were less than 30 years of age, and only 7.5% of the workers (31) were of age 50 years or more. A majority of the workers were males (85%). The mean age of the male workers was 35.4 years and for the female workers was 40.8 years. Around 14% (60) of the workers had an experience of five years or less, whereas, a majority of them had (86%) more than five years experience. The total number of accidents was 77, of which the majority (65) were males and the others were females.

The overall prevalence rate of accidents was found to be 18.5%. The prevalence rate of accidents was highest among the below-20-year age group. It was significantly higher than the prevalence rate in other age groups (*P* < 0.05). As the age increased, the prevalence rate decreased. Males and females showed an almost similar pattern of accidents. The difference in prevalence rate of accidents seen in males and females was not statistically significant (χ^2^ = 0.06, *P* > 0.05). The prevalence rate was lowest among those with more than five years of job experience (16%) compared to those with less than five years of job experience (33.3%). The lowest prevalence in this group was found to be statistically significant (χ^2^ = 10.2, *P* = 0.001). Workers having lesser experience (OR = 2.62: 95% CI, 1.37-5.01) were at an increased risk of accident occurrence [Table 1].

**Table 1:** Prevalence of accidents according to age, sex, and job duration

	Number of subjects	Number of accidents	Prevalence (%)	χ^2^, *P* value
Age group				
<20	10	4	40.0	12.03, 0.02*
20-29	65	18	27.7	
30-39	160	33	20.6	
40-49	150	18	12.0	
50 and more	31	4	5.2	
Sex				
Male	355	65	18.3	0.06, 0.8
Female	61	12	19.7	
Job duration (in years)				
<5 years	60	20	33.3	10.21, 0.001*
5 years and above	356	57	16.0	

* *P* value < 0.05 is considered as significant

On an individual analysis of accidents, according to the body parts involved in the accidents, it was found that almost 86% of the accidents had affected the limbs (upper limb 25%, lower limb 61%). Trunk affection was seen in 7.8% of the accidents and head and neck had been affected in 2.6% of the accidents [Table 2]. When the accidents were analyzed according to the type of injuries [Table 3], it was found that superficial injuries like cuts, abrasions, and so on, contributed to the highest number of injuries (52%). Sprain-strain and bony injuries comprised of 35.1% and 10.4% of all the accidents, respectively. So far as the incidents that had occurred during the accidents are concerned, the tabulation shows [Table 4] that about 20.8% of the accidents had each been caused by the fall of an object or striking against an object. Furthermore, 19.5% and 18.1% of accidents are due to handling and fall of a person, respectively. Other causes were hand tools (9.1%), machinery in motion (10.4%).

**Table 2:** Distribution of accidents according to body parts involved *n* = 77

Body parts involved	Number	Percentage
Head and neck	2	2.6
Trunk	6	7.8
Upper limb	19	24.7
Lower limb	47	61
Testis	1	1.3
Superficial burn	1	1.3
Multiple abrasion	1	1.3

**Table 3:** Distribution of accidents according to type of injury *n* = 77

Type of injury	Number	Percentage
Superficial injury	40	51.9
Sprain and strain	27	35.1
Bony injury	8	10.4
Burn	1	1.3
Eye injury	1	1.3

**Table 4:** Distribution of accidents according to incidents *n* = 77

Incident	Number	Percentage
Handling	15	19.5
Fall of a person	14	18.1
Fall of an object	16	20.8
Striking against objects	16	20.8
Hand tools	7	9.1
Machinery in motion	8	10.4
Burns	1	1.3

Around 44% of the accidents (34) occurred before 12 o'clock noon and 46% between 12 o'clock noon and 4 pm in the day [Table 5]. Absenteeism due to accidents was four to 13 days among nearly half of the injured workers (36,47%), followed by less than three days (25, 32.5%), 21 days and more (11, 14.3), and 14 to 20 days (5, 6.5%). Multiple logistic regression analysis revealed that the 30-39 years age group had an independent significant association with accidents (Adjusted Odds Ratio = 0.21 (0.05-0.97), *P* = 0.04) [Table 6].

**Table 5:** Time in the day of the accident *n* = 77

Time of the day	Numbers	Percentage
8 am to 10 am	14	18.2
10 am to 12 noon	20	26.0
12 noon to 2 pm	18	23.4
2 pm to 4 pm	17	22.1
4 pm to 6 pm	6	7.8
Others	2	2.6

**Table 6:** Correlates of accidents: Multiple logistic regression analysis

Variables	Odds ratio unadjusted (95% CI)	Odds ratio adjusted (95% CI)	*P* value
Age group			
>50	-	-	-
40-49	0.92 (0.28-2.94)	0.51 (0.13-2.00)	0.34
30-39	1.75 (0.57-5.36)	0.21 (0.05-0.97)	0.04*
20-29	2.58 (0.79-8.43)	0.36 (0.04-2.90)	0.34
<20	4.50 (0.87-23.29)	0.39 (0.03-4.18)	0.43
Sex			
Female	-	-	-
Male	0.93 (0.46-1.82)	2.66 (0.96-7.31)	0.06
Job duration (years)			
>5	-	-	-
<5	2.62 (1.37-5.01)	0.33 (0.07-1.62)	0.17

* *P* value < 0.05 is considered as significant

## Discussion

Well-documented studies to determine the prevalence rate and pattern of accidents in tile factories are very few at the global level. There are no studies to know the prevalence rate and pattern of accidents in tile factories in India. However, there are some accident-related studies in other factories. The overall prevalence rate of accidents in tile factories was 18.5%. It was found that almost around 86% of the accidents had affected the limbs (upper limb 24.7%, lower limb 61%), superficial injuries like cuts, abrasions, and so on, contributed to a majority of the injuries (52%), 40% of the accidents were due to stepping/striking against objects and while handling. Hand tools and machinery in motion contributed to around 20% of the accidents. So far as the age group was concerned, age < 20 years was found to have an increased risk of accident causation and it was found to be statistically significant (χ^2^ = 12.03, *P* = 0.02). Workers having lesser experience (χ^2^ = 10.21, *P* = 0.001, OR = 2.62) were at an increased risk of accident occurrence.

A study done in a metal smelting industry showed around 28% accidents during a five-year period, from 2000 to 2004.(8) Another study in a garment industry showed incidence of accidents during a three-month period to be as little as 2.49/1000 workers.(9) The high prevalence of accidents in our study may be due to lack of awareness and training given to the workers, coupled with the involvement of young and less-experienced workers. Workers in tile industries will be stressed from excess heat and humidity and fumes and dust that often cause a strain, which is reflected in terms of an increase in the number of accidents. Also the workers change their duty frequently from one type of job to another. Other studies have also shown that accidents are more common among the younger age group and less-experienced workers.(10–12) A high proportion of superficial injuries is mainly due to stepping/striking against objects, and frequent association with handling of small tools, which is in comparison with another study.(8) Our study shows that a majority of the accidents took place before 2 pm in the day in contrast to the above-mentioned study.(8) This again illustrates the difficulty in adjustment to work due to lack of training or experience. However, the adjusted Odds Ratio for the 30-39 years age group revealed that there was a 79% reduction in the incidence rate of accidents in this age group as compared to the above 50 years age group. This might probably be due to an increase in working efficiency in this age group.

The record-based, cross-sectional nature and relatively smaller sample size were the limitations of the study. Because of feasibility constraints we could not include the tile factories outside Mangalore city area. There might be a possibility of an underestimation of the true prevalence because of non-reporting of mild injuries. Also some of the information was not present in the records, such as, smoking, chewing habits, drug addictions, and other related factors. Longitudinal studies will be required to further look for an association between various factors associated with accidents. Accidents in tile industries are an important occupational health problem in this area of the country, in the context of a high prevalence rate of accidents and larger involvement of the at-risk worker population in tile factories. Our study has demonstrated that a majority of the injuries are due to human error in these accidents and indicates the necessity of proper safety training for the workers. Various studies highlighted the importance of safety measures to prevent accidents in various sectors.(13–15) This study has categorized some high-risk groups such as young and less-experienced workers. These worker groups need special attention so far as workplace safety is concerned.
